# Dl-3-n-Butylphthalide Reduces Cognitive Deficits and Alleviates Neuropathology in P301S Tau Transgenic Mice

**DOI:** 10.3389/fnins.2021.620176

**Published:** 2021-02-10

**Authors:** Yanmin Chang, Yi Yao, Rong Ma, Zemin Wang, Junjie Hu, Yanqing Wu, Xingjun Jiang, Lulu Li, Gang Li

**Affiliations:** ^1^Department of Neurology, Union Hospital, Tongji Medical College, Huazhong University of Science and Technology, Wuhan, China; ^2^Department of Pharmacology, Tongji Medical College, Huazhong University of Science and Technology, Wuhan, China; ^3^Harvard Medical School, Boston, MA, United States

**Keywords:** Dl-3-n-butylphthalide, tau, P301S, cognitive deficits, neuroinflammation

## Abstract

Alzheimer’s disease (AD) is a destructive and burdensome neurodegenerative disease, one of the most common characteristics of which are neurofibrillary tangles (NFTs) that are composed of abnormal tau protein. Animal studies have suggested that dl-3-n-butylphthalide (dl-NBP) alleviates cognitive impairment in mouse models of APP/PS1 and SAMP8. However, the underlying mechanisms related to this remain unclear. In this study, we examined the effects of dl-NBP on learning and memory in P301S transgenic mice, which carry the human tau gene with the P301S mutation. We found that dl-NBP supplementation effectively improved behavioral deficits and rescued synaptic loss in P301S tau transgenic mice, compared with vehicle-treated P301S mice. Furthermore, we also found that it markedly inhibited the hyperphosphorylated tau at the Ser262 site and decreased the activity of MARK4, which was associated with tau at the Ser262 site. Finally, dl-NBP treatment exerted anti-inflammatory effects and reduced inflammatory responses in P301S mice. In conclusion, our results provide evidence that dl-NBP has a promising potential for the therapy of tauopathies, including AD.

## Introduction

Alzheimer’s disease (AD), the foremost cause of dementia in the elderly, is a complex multi-factorial neurodegenerative disease. It is characterized by two classic neuropathological hallmarks: senile plaques consisting of a progressive accumulation of amyloid-β peptide (Aβ) and neurofibrillary tangles (NFTs). NFTs are formed by the aggregation of paired helical filaments (PHFs) composed of truncated and hyperphosphorylated microtubule-associated protein tau (Arai et al., 1990; Braak and Braak, 1991; Citron, 2010). The latter type of lesion characterizes a variety of human neurodegenerative diseases collectively termed “tauopathies” (Murray et al., 2014; Kovacs, 2015), including AD, progressive supranuclear palsy, corticobasal degeneration, and Pick’s disease (Spillantini and Goedert, 2013; Wang and Mandelkow, 2016). The etiologies of tauopathies are not completely understood. A series of transgenic mice that express human mutations of tau proteins have been developed to investigate the role of tau in tauopathies. P301S is a mouse model that overexpresses the human tau gene with the P301S mutation in exon 10. It expresses about fivefold greater human tau than endogenous mouse tau, but no amyloid-beta (Aβ) peptide deposition. Furthermore, it is accompanied by increased microglial activation, synaptic deficits, and behavioral impairments (Bellucci et al., 2004; Yoshiyama et al., 2007; Scattoni et al., 2010; Dumont et al., 2011).

Dl-3-n-butylphthalide (dl-NBP) was first isolated from the seeds of Chinese celery. It is a synthetic chiral compound that contains the L- and D-isomers of butylphthalide. In 2002, this compound was approved for use with stroke patients by the State Food and Drug Administration of China. Some evidence has revealed that the administration of dl-NBP ameliorates oxidative stress and pro-mitochondrial function, decreases neuronal apoptosis, and inhibits the inflammatory responses in experimental ischemic models (Li et al., 2010; Liu et al., 2017; Tian et al., 2017; Zhou et al., 2019). Furthermore, dl-NBP has been shown to have a protective effect on vascular cognitive impairment (Liao et al., 2009; Jia et al., 2016; Qi et al., 2018). Recently, studies have shown that dl-NBP treatment plays a protective role in decreasing Aβ levels, rescuing neuronal losses, inhibiting neuroinflammatory responses, reducing oxidative injuries, and alleviating learning and memory impairments in some AD models (Peng et al., 2007, 2009, 2010, 2012). However, there has been no specific study on the interaction between dl-NBP and tauopathy.

Herein, we studied the role of dl-NBP in an AD transgenic mouse model for AD-related tauopathy, which expresses the human tau gene with the P301S mutation. We found that the administration of dl-NBP significantly decreased the levels of hyperphosphorylated tau at Ser262 in P301S mice, compared with vehicle-controlled mice. Furthermore, treatment with dl-NBP significantly improved synaptic integrity, reduced neuroinflammation, and ultimately ameliorated cognitive impairment in P301S mice, compared with vehicle-treated P301S mice.

## Materials and Methods

### Animals and Drug Administration

P301S transgenic mice [B6; C3-Tg (Prnp-MAPT^∗^P301S)/Nju] that express human mutant microtubule-associated protein tau (Yoshiyama et al., 2007) were purchased from the Model Animal Research Center of Nanjing University (No. 32002100001876). The experimental mice were raised in a standard specific-pathogen-free (SPF) animal house (No. 00187090). This type of transgenic mouse has a C57Bl/6J background. All transgenic and non-transgenic mice used in this study were littermates of P301S mice. The mice were allowed free access to food and water. All treatments were approved by the Institutional Animal Care and Use Committee at Tongji Medical College, Huazhong University of Science and Technology (2017, IACUC No. 2364).

Dl-NBP was provided by CSPC NBP Pharmaceutical Co., Ltd. (Shijiazhuang, Hebei, China). Dl-NBP was used for oral management at 30 mg/kg body weight, as in previous studies (Yang et al., 2015; Wang et al., 2016). Eleven P301S mice were administered 30 mg/kg dl-NBP by intragastric administration three times a week for 16 weeks from 4 months old (group: P301S + dl-NBP). Meanwhile, 11 non-transgenic mice (group: WT) and 11 P301S mice (group: P301S) were administered vegetable oil in the same manner without dl-NBP three times a week for 16 weeks from 4 months old. The weights of the mice were recorded weekly to determine the appropriate drug dosage for the mice. Behavioral tests were performed 16 weeks after intragastric administration, after which the mice were anesthetized and dissected.

### Morris Water Maze

For spatial learning and memory measurement, we performed the Morris water maze (MWM) test (Morris et al., 1982). For the MWM test, we used a circular container with a radius of approximately 75 cm, on top of which was a tracking system. The test was conducted over 7 days. The acquisition period lasted for the first 6 days. The swimming paths and latencies (time spent to find the platform) of the mice were recorded. Mice started the test from one of four quadrants, facing the wall of the pool. The mice had to swim to a hidden underwater platform. If a mouse swam onto the hidden platform within 1 min, the time the mouse spent to swim there was recorded. If a mouse failed, the mouse was guided to the platform and stayed on it for 20 s. Data were recorded using a video camera located 1.5 m above the water. The camera was connected to a digital tracking device attached to an IBM computer. The probe trial was performed on the seventh day. During this period, the platform was removed, and the mice were allowed to swim freely for 1 min. Data regarding the traveling times the mice spent crossing the platform area and their time spent in each quadrant were collected.

### Open-Field Test

We performed an open-field test (OFT) in a square box (45 × 45 cm). The environment was kept quiet. Before the test began, each mouse was allowed to adapt to the open-field arena for 30 min. Then, the mice were placed in the center of the box. A computer-assisted infrared tracking system recorded the mouse for 5 min. The times spent in the center and peripheral zones were recorded. In addition, the total distance traveled was used to measure locomotor activity. To avoid the influence of odor in the wooden box, the box was wiped with a solution of 70% ethanol.

### Fear Conditioning Test

Mice were placed in a square box (25 × 25 cm; Coulbourn Instruments, Holliston, MA, United States) that included a metal floor and a sound system. First, a test mouse was placed in the box for 3 min to adapt to the environment. Then, during the training session, the mouse was given an auditory cue of 70 dB for 30 s, followed by a mild foot shock (0.5 mA for 2 s). The operation was repeated twice with 2 min intervals. For the short-term memory test, the mice were placed in the chamber again without stimulus for 5 min after 4 h of the training session, and the “freezing” time was recorded for a memory assessment. For the 24 h long-term memory test, the process was repeated. A camera and a circuit board were connected to a personal computer, and software (Freeze Frame 2.0; Actimetrics) controlled the circuit and recorded data.

### Electrophysiological Analysis

Mice were decapitated after being deeply anesthetized. The brains of the mice were removed and put into oxygenated ice-cold artificial cerebrospinal fluid (aCSF) that was composed of 124 mM NaCl, 2 mM KCl, 1.25 mM NaH_2_PO_4_, 26 mM NaHCO_3_, 2 mM MgSO_4_, 2.5 mM CaCl_2_, and 10 mM glucose (pH 7.4; 305 mOsm). Coronal brain slices (300 μm thick) were cut at 4°C in the aCSF using a Leica VT1000S vibratome (Milton Keynes, United Kingdom) and saturated with carbogen (95% O_2_ and 5% CO_2_). After slicing, sections were immediately transferred to an incubation chamber filled with oxygenated aCSF and were equilibrated at room temperature for 30 min before recording. Slices were placed in a chamber and recorded with an 8 × 8 microelectrode array at the bottom (Parker Technology, Beijing, PRC) and kept submerged in aCSF. Signals were detected using a MED64 System (Alpha MED Sciences, Osaka, Japan). We recorded the field excitatory postsynaptic potentials (fEPSPs) in the CA1 region by stimulating CA3 neurons. We used the current that corresponded to a maximum amplitude of 40% of the induced fEPSPs as the appropriate electrical stimulation. The baseline was recorded for 30 min, followed by high-frequency stimulation (HFS: 100 Hz, 1 s duration). Then, long-term potentiation (LTP) data continued to be recorded for 1 h. The magnitudes of LTP were quantified as the mean percentage of baseline fEPSP initial slope.

### Western Blot Analyses

After the behavioral test, half of the mice were randomly selected from the three groups and anesthetized. The cortexes and hippocampus of these mice were removed and stored at −80°C for biochemical studies. Protein samples were extracted, and their concentrations were measured. The protein samples were separated on a 10 or 12% SDS-PAGE gel. Then, they were transferred onto polyvinylidene fluoride (PVDF) membranes or nitrocellulose (NC) membranes, followed by blocking in 5% non-fat milk for 1.5 h at room temperature. The membranes were then incubated with primary antibody (Table 1) at 4°C overnight. After washing, the membranes were incubated with horseradish peroxidase (HRP)-labeled secondary antibody for 1 h at 25°C. Secondary antibodies used for western blot purposes were anti-rabbit IgG (1:3,000; Cell Signaling Technology) and anti-mouse IgG (1:5,000; Proteintech). Enhanced chemiluminescence (ECL) kits were used to dye the membranes, and bands were detected using an exposure instrument (Bio-Rad, Richmond, CA, United States).

**TABLE 1:** The following primary antibodies were used for western blotting.

**Antibody**	**Host**	**Dilution**	**Source**
GSK3β	Rabbit	1:1,000	Cell Signaling technology
Phosphor-GSK3β(Ser9)	Rabbit	1:1,000	Cell signaling technology
CDK5	Rabbit	1:1,000	Cell signaling technology
p35/25	Rabbit	1:1,000	Cell signaling technology
Phosphatase 2A C subunit	Rabbit	1:1,000	Cell signaling technology
Phosphor-PP2A (Y307)	Rabbit	1:1,000	R&D Systems
MARK4	Rabbit	1:1,000	Cell signaling technology
PSD95	Rabbit	1:2,000	Cell signaling technology
NR2B	Rabbit	1:1,000	Proteintech
GluR1	Rabbit	1:1,000	Cell signaling technology
GFAP	Rabbit	1:1,000	Proteintech
IL-1β	Rabbit	1:1,000	Proteintech
TNFα	Rabbit	1:500	Proteintech
p-Tau (Ser199)	Rabbit	1:2,000	Signalway antibody
Tau5	Mouse	1:1,000	Abcam
p-Tau (Ser262)	Rabbit	1:1,000	Proteintech
p-Tau (Ser396)	Rabbit	1:1,000	Sigma
p-Tau (Ser404)	Rabbit	1:2,000	Sigma
p-Tau (Thr231)	Rabbit	1:1,000	Signalway antibody
p-Tau (Thr205)	Rabbit	1:1,000	Signalway antibody
β-Actin	Mouse	1:5,000	Proteintech
LC3	Rabbit	1:1,000	Proteintech
P62	Rabbit	1:1,000	Proteintech
DM1A	Mouse	1:2,000	Cell signaling technology

### Immunohistochemistry

After the behavioral test, half of the mice were randomly selected from the three groups and anesthetized. Then, they were perfused with sodium chloride and paraformaldehyde. After, their brains were removed, immersed in paraformaldehyde, and embedded in paraffin. Brain slices were cut coronally at a thickness of 5 μm. The slides were dewaxed, and antigen was repaired (high temperature and high pressure conditions were used to repair the tissue section) and incubated with the corresponding primary antibody (Table 2) overnight at 4°C. After washing, the sections were incubated with a secondary antibody for 1 h at 25°C. The sections were washed again and reacted in 3,3′-diaminobenzidine (DAB) and counterstained with hematoxylin. Finally, the slices were sealed. Images were collected using an Olympus photomicroscope (Nikon, 90i, Tokyo, Japan).

**TABLE 2:** The following primary antibodies were used for immunohistochemistry.

**Antibody**	**Host**	**Dilution**	**Source**
p-Tau (Ser262)	Rabbit	1:200	Proteintech
GFAP	Rabbit	1:200	Sigma
CD11b	Rabbit	1:200	Sigma

### Nissl Staining

Brain slices were deparaffinized in xylene and rehydrated in ethanol. These were successively passed in different concentrations of ethanol of 100, 95, 90, and 70%, each for 5 min. The brain slices were then stained with Nissl solution for 3 min and cleaned with distilled water for 10 s. This was followed by passing in dimethylbenzene for 5 min. The brain slices were then sealed with neutral balsam. Finally, images were collected using an Olympus photomicroscope (Nikon, 90i, Tokyo, Japan).

### Statistical Analysis

All statistical results are expressed as means ± SEMs. We employed one-way and two-way ANOVAs for comparisons between the three groups. Data were analyzed using GraphPad Prism 7.0 software. Comparing P301S with WT mice, results were considered to have significant differences at *P* < 0.05. Meanwhile, comparing P301S with dl-NBP-treated P301S mice, results were considered to have significant differences at *P* < 0.05.

## Results

### dl-NBP Treatment Ameliorates Behavioral Deficits in P301S Mice

**FIGURE 1:**
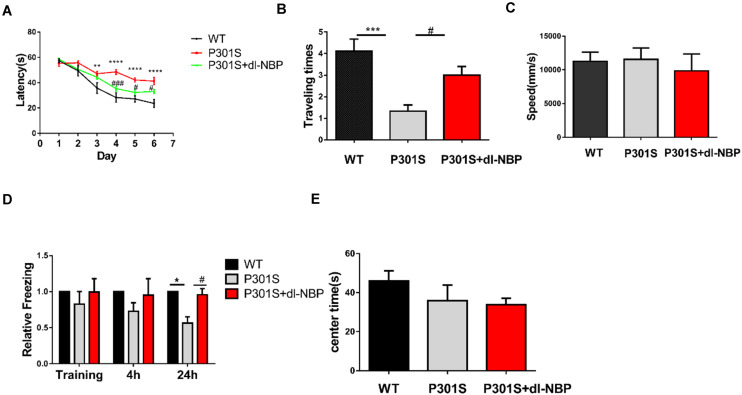
dl-NBP treatment ameliorates behavioral deficits in P301S mice. **(A)** Escape latency to reach the platform in the training phase of the Morris water maze (MWM) over 6 consecutive days. The spatial learning ability of P301S mice was declined compared with WT mice, and it was alleviated in P301S + dl-NBP mice. **(B)** The numbers of entry to the platform zone of the three groups in the probe trial of the Morris water maze test. **(C)** Swimming speed to test the motor ability of the three groups of mice in the Morris water maze test. **(D)** Relative freezing time at 4 and 24 h during contextual fear conditioning test. dl-NBP ameliorated contextual memory deficits at 24 h during contextual fear conditioning test in P301S mice. **(E)** Time spent in the center of the open field during the open-field exploration of the three groups. All results were presented as mean ± SEM (*n* = 8). *P < 0.05, **P < 0.01, ***P < 0.001, ****P < 0.0001, WT mice vs. P301S mice; **^#^***P* < 0.05, **^###^***P* < 0.001, P301S mice vs. P301S + dl-NBP mice.

Mice were treated with either dl-NBP or vegetable oil at 4 months old for 4 months. To evaluate whether dl-NBP treatment could ameliorate behavioral deficits in P301S mice, we conducted a MWM test, OFT, and fear conditioning test. The experimental methods involved in our study are described above. The MWM test is one of the most widely recognized behavioral tests used to assess hippocampal-dependent spatial learning and memory. Spatial learning was assessed using escape latency (time spent to find the hidden platform). As shown in Figure 1A, all mice showed decreased escape latency in the training phase. P301S mice performed significantly worse on finding the underwater platform than WT mice, and this phenomenon partly improved in dl-BP-treated P301S mice. In the probe trial, removing the escape platform, we detected the beneficial effects of dl-NBP treatment on spatial memory. The P301S mice treated with dl-NBP showed significantly more time to enter and cross the target platform region (Figure 1B) than the vehicle-treated P301S mice. In addition, similar swimming speeds were observed among the three groups (Figure 1C), indicating that the athletic ability of the mice did not differ. The contextual fear conditioning test is another test used to measure memory. All mice underwent this test. The results showed an increased freezing time at 24 h in NBP-administered P301S mice, compared with vehicle-treated P301S mice (Figure 1D), indicating that dl-NBP treatment improved long-term memory in P301S mice. In addition, we performed OFT, and we observed that dl-NBP treatment did not significantly change the time to the center of the field, suggesting that there was no anxiety-related phenotype between these three groups (Figure 1E).

In summary, the above results provide evidence that treatment with dl-NBP can effectively improve learning and spatial memory in P301S mice.

### dl-NBP Treatment Reduces Hyperphosphorylation of Tau at Ser262 in P301S Mice

**FIGURE 2:**
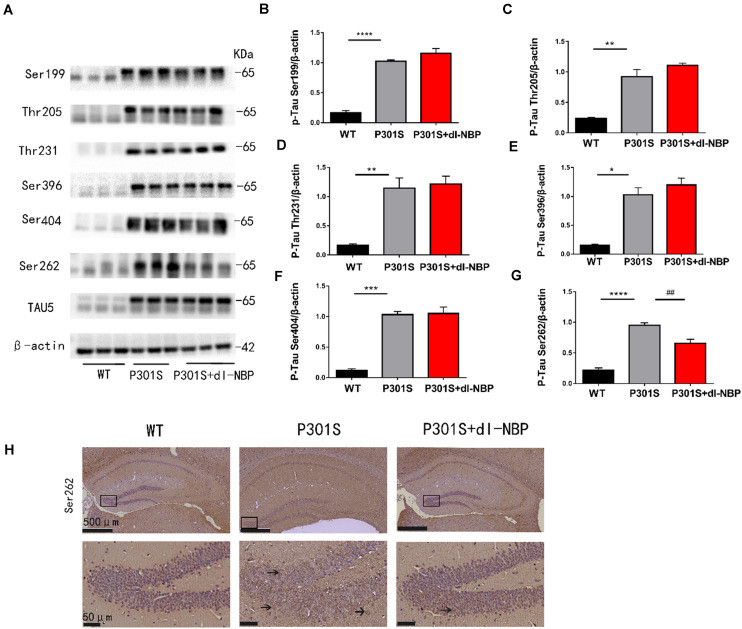
dl-NBP treatment reduced hyperphosphorylation of tau at Ser262 in P301S mice. **(A)** Western blot analysis of phosphorylated tau at residues Ser396, Ser404, Ser199, Ser262, Thr231, and Thr205 in the brains. β-Actin served as a loading control. **(B–G)** Quantitative analysis of tau phosphorylation levels at the Thr205, Ser396, Ser404, Ser199, Ser262, and Thr231 sites. All results are presented as mean ± SEM (*n* = 3). *P < 0.05, **P < 0.01, ***P < 0.001, ****P < 0.0001, WT mice vs. P301S mice; **^##^***P* < 0.01, P301S mice vs. P301S + dl-NBP mice. **(H)** Detection of tau phosphorylation in the hippocampi by immunohistochemistry with anti-phospho-tau (Ser262) (scale bars: upper panel—500 μm, lower panel—50 μm).

Elevated levels of hyperphosphorylated tau are a major pathologic feature in AD. Thus, we conducted western blotting and immunohistochemistry to investigate whether dl-NBP treatment could decrease hyperphosphorylated tau in P301S mice. As shown in Figures 2A–G, there were no significant changes in the levels of hyperphosphorylated tau at Ser396, Ser404, Thr205, Ser199, and Thr231. Intriguingly, treatment with dl-NBP resulted in a remarkable reduction in the levels of hyperphosphorylated tau at Ser262 in P301S mice, compared with vehicle controls. Next, we determined the levels of hyperphosphorylated tau at Ser262 using immunohistochemistry. The result was consistent with the western blot (Figure 2H).

Taken together, these results showed that dl-NBP treatment evidently reduced tau hyperphosphorylation at Ser262 in the hippocampi of P301S mice.

### dl-NBP Rescues Synaptic Loss in P301S Mice

**FIGURE 3:**
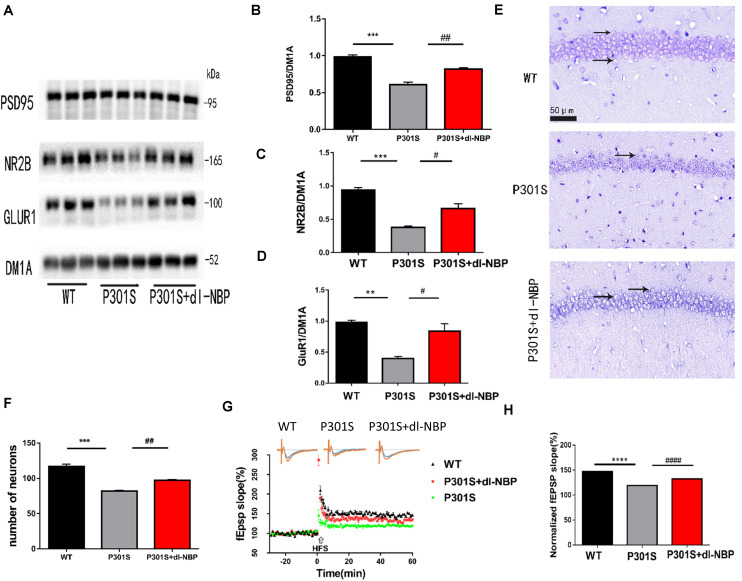
dl-NBP rescued synaptic loss in P301S mice. **(A)** Representative western blots of synaptic plasticity-related proteins GLUR1, NR2B, and PSD95 of the hippocampus from three groups. DM1A was shown as a loading control. **(B–D)** Quantitative analysis of GLUR1, NR2B, and PSD95/DM1A ratio. **(E)** Representative Nissl staining sections of the hippocampal CA1 region. Scale bars: upper panel—500 μm, lower two panels—50 μm. **(F)** Quantitative analysis of surviving neurons in the region of CA1. **(G,H)** The slope of fEPSP after high-frequency stimulation (HFS) recorded on hippocampal slices of the three groups. LTP magnitude was calculated as the average (normalized to baseline) of the responses recorded 40–60 min after conditioning stimulation. All results were presented as mean ± SEM (*n* = 5). **P < 0.01, ***P < 0.001, ****P < 0.0001, WT mice vs. P301S mice; **^#^***P* < 0.05, **^##^***P* < 0.01, **^####^***P* < 0.0001, P301S mice vs. P301S + dl-NBP mice.

To examine whether dl-NBP helped improve the synaptic function of P301S mice, we assessed the levels of several synapse-related proteins (Figures 3A–D). α-Amino-3-hydroxy-5-methyl-4-isoxazolepropionic acid (AMPA) is a glutamate receptor. The changes in the number and composition of AMPA-type glutamate receptors (AMPARs) in the postsynaptic membrane have been shown to have an impact on the expression of LTP. At excitatory synapses, N-methyl-D-aspartate receptors (NMDARs) play an important role in shaping synaptic responses acutely and in the induction of LTP. Hence, we examined the protein levels of the NMDAR obligatory subunit NR2B and the AMPAR subunit GluR1. Post-synaptic density protein 95 (PSD95) is also a critical synaptic protein. It is important for synaptic plasticity. Its level is diminished in AD. The data showed that protein levels of the synaptic proteins (NR2B, GluR1, PSD-95) in the hippocampus were lower in P301S mice than in WT mice. Interestingly, the protein levels were elevated in P301S mice treated with dl-NBP, compared with vehicle-treated P301S mice. Nissl staining is a method of dyeing nerve tissue with basic dyes. As shown in Figures 3E,F, P301S mice showed obvious neuronal death in the hippocampi, compared with WT mice. Treatment with dl-NBP had an obvious protective role and decreased neuronal death.

Synaptic plasticity is the premise of learning and memory. One of its primary manifestations is LTP. As we wondered whether dl-NBP improved synaptic plasticity in P301S mice, we utilized acute brain slices from three groups of mice and electrorecorded hippocampal slices to examine the LTP of the CA3-to-CA1 region (Figures 3G,H). FEPSPs revealed a significant decrease in P301S mice, compared with WT mice. At the same time, there was an increase in P301S + dl-NBP mice, indicating that administering dl-NBP helped to upregulate synaptic plasticity in P301S mice.

Hence, the results suggested that dl-NBP has the potential to rescue the impairment in synaptic function in P301S mice.

### dl-NBP Ameliorates Tau Phosphorylation *via* MARK4 in P301S Mice

**FIGURE 4:**
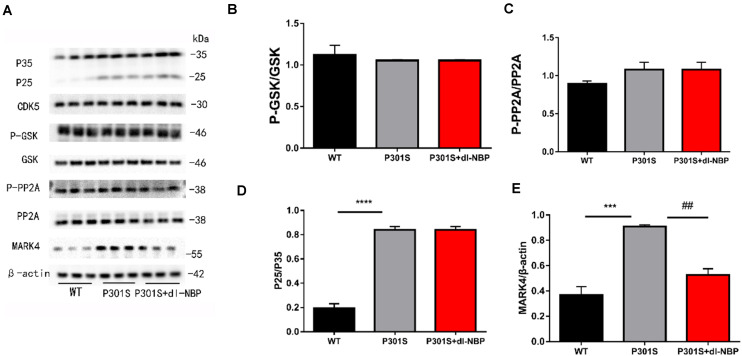
dl-NBP ameliorates tau phosphorylation *via* MARK4 in P301S mice. **(A)** Western blotting of P25-35, CDK5, PP2A, GSK-3β, P-GSK-3β, and MARK4. β-Actin served as the loading control. **(B)** Quantitative analysis of P25/P35 ratio. **(C)** Quantitative analysis of PP2A/P-PP2A ratio. **(D)** Quantitative analysis of GSK-3β/P-GSK-3β ratio. **(E)** Quantitative analysis of MARK4/β-actin. All results were presented as mean ± SEM (*n* = 4). ***P < 0.001, ****P < 0.0001, WT mice vs. P301S mice; **^##^***P* < 0.01, P301S mice vs. P301S + dl-NBP mice.

As GSK3β, CDK5, PP2A, and MARK4 have been linked to tau hyperphosphorylation, we next explored the expression of these kinases and phosphatases after treatment with dl-NBP. GSK-3β increases p-tau levels. Phosphorylation of GSK-3β at Ser9 decreased GSK-3β activity. PP2A helped to dephosphorylate tau. The phosphorylation of PP2A at Tyr307 was found to inactivate PP2A and increase hyperphosphorylated tau, finally causing NFTs. As shown in Figure 4A, the ratio of p-GSK3β/GSK3β, P-PP2A/PP2A, and P25/P35 between the P301S and P301S + dl-NBP groups did not differ significantly (Figures 4B–D). However, the results of western blotting showed that dl-NBP treatment in P301S mice notably reduced the expression of MARK4, compared with P301S mice (Figure 4E). Accumulating evidence suggests that p-tau may affect the flux of autophagy. We thus utilized western blotting to detect the autophagy marker proteins LC3II/I and P62. However, no differences were detected in LC3II/I and P62 levels between the groups of P301S and P301S + dl-NBP.

### dl-NBP Treatment Inhibits Inflammation in P301S Mice

**FIGURE 5:**
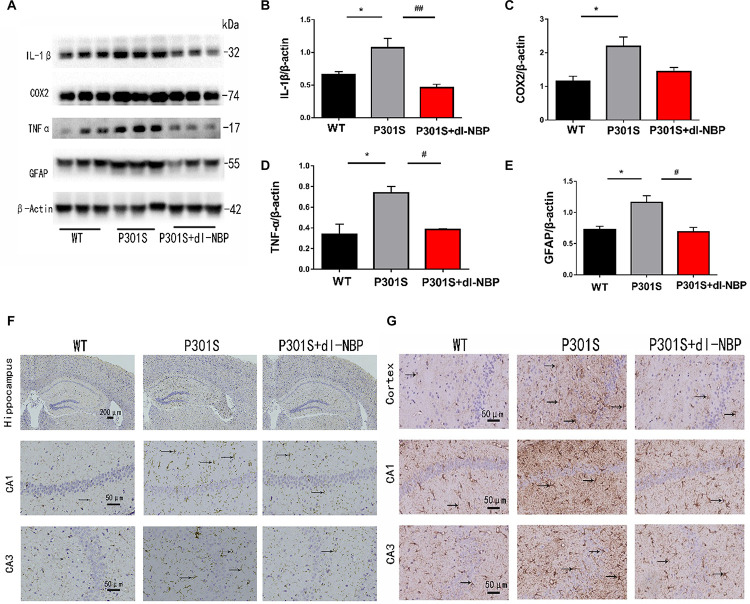
dl-NBP treatment inhibited inflammation in P301S mice. **(A)** Western blotting of GFAP, IL-1β, TNFα, and cox2. **(B–E)** Quantitative analyses of western blot for GFAP, IL-1β, TNFα, and COX2. β-Actin was used as an internal control. **(F)** Immunohistochemical staining with CD11b antibody and the GFAP antibody in the brains of the three groups of mice. Scale bars: upper panel—200 μm, lower two panels—50 μm. **(G)** Immunohistochemical staining GFAP antibody in the brains of three groups of mice. Scale bar: 50 μm. All results were presented as mean ± SEM (*n* = 4). *P < 0.05, WT mice vs. P301S mice; **^#^***P* < 0.05, **^##^***P* < 0.01, P301S mice vs. P301S + dl-NBP mice.

To assess whether dl-NBP helped reduce neuroinflammation in P301S mice, we next measured the levels of inflammatory markers. As shown in Figures 5A–E, dl-NBP treatment in P301S mice significantly decreased the expression of glial fibrillary acidic protein (GFAP), tumor necrosis factor-α (TNF-α), and interleukin-1β (IL-1β) in the hippocampi of vehicle-treated P301S mice. In addition, we explored the immunoreactivity of cluster of differentiation 11b (CD11b; a marker of microglial cell activation) and GFAP (a marker of astrocytes). As shown in Figures 5F,G, the administration of dl-NBP decreased the levels of both CD11b and GFAP in the brains of P301S mice, compared with control P301S mice.

Taken together, these results demonstrate that the administration of dl-NBP reduces neuroinflammation in P301S mice.

## Discussion

AD is the leading cause of dementia among the elderly. As clinical trials that target anti-Aβ therapies have failed to inhibit the progression of AD patients’ cognitive decline, tau has been considered to have a good prospect as a therapeutic target. As a drug for stroke treatment in China, dl-NBP has been reported to ameliorate cognitive impairment in vascular dementia. In addition, it has been shown to significantly improve cognitive functioning in AD models that target Aβ. The P301S mouse, which carries the human tau gene with the P301S mutation, is a well-characterized mouse model that is used to investigate effective treatment strategies for tauopathies. This study aimed to investigate the potential protective role of dl-NBP against cognitive decline in P301S transgenic mice.

We demonstrated that chronic gastric perfusion for 16 weeks of dl-NBP reduced cognitive deficits in P301S mice. In the MWM behavioral test, we observed that treatment with dl-NBP remarkably ameliorated the spatial learning and memory impairments in P301S mice, as deduced by the shorter escape latencies observed in the training phase and the number of entries into the platform zone during the probe trial. Moreover, as a second measure to assess memory, the contextual fear conditioning test revealed that dl-NBP treatment improved long-term memory, as shown by an obviously increased freezing time during the memory test at 24 h. As learning and memory are regulated by synaptic function and integrity, our behavioral test results suggest that treatment with dl-NBP could play a protective role for synaptic deficiency in P301S mice.

Synaptic plasticity is a functional term that describes alterations in synaptic efficiency. It is thought to be the biological foundation of learning and memory. Cognitive decline among the aged is considered to be related to a decrease in structural and functional plasticity in the hippocampi. LTP is an important form of synaptic plasticity (Malenka and Bear, 2004; Wu et al., 2019). We examined LTP in the treated mice. A higher fEPSP was induced in P301S mice treated with dl-NBPl in our study, compared with age-matched vehicle-treated P301S mice. At the same time, we uncovered an alleviation in synaptic potentiation in dl-NBP-administered P301S mice. We also examined the protein levels of well-established synaptic markers, including the NMDAR obligatory subunit NR2B, the AMPAR subunit GluR1 (Huganir and Nicoll, 2013; Diering and Huganir, 2018; Li et al., 2019), and PSD95 in the hippocampi (Hoover et al., 2010; Sydow et al., 2011) using western blotting. We observed that compared with the P301S group, the treatment of dl-NBP in P301S mice increased the protein levels of the synaptic markers above, which may contribute to the increase of neurons in the hippocampi. These results suggest that dl-NBP effectively improves neuronal loss and synaptic dysfunction in P301S mice.

The accumulation of hyperphosphorylated tau is evident in P301S mice. We explored whether dl-NBP affects the regulation of hyperphosphorylated tau. In previous studies, hyperphosphorylated tau had been found in more than 40 serine/threonine residues from various neurodegeneration patients (Wang and Liu, 2008; Avila, 2009; Hanger et al., 2009). We selectively examined several sites of hyperphosphorylated tau that may correlate with severe pathology. The results of our examinations revealed that in P301S mice, the levels of hyperphosphorylated tau at the Thr205, Ser396, Ser404, Ser199, and Thr231 sites were not evidently decreased by treatment with dl-NBP. Intriguingly, there was remarkable inhibition at the Ser262 site. This site is located in the KXGS motif in one of the microtubule-repeat domains. Hyperphosphorylated tau at Ser262 is an earlier pathological change and plays an initiating role in abnormal metabolism and accumulation of tau (Nishimura et al., 2004; Ando et al., 2016; Lu et al., 2016). Blocking tau phosphorylation at this site can effectively decrease tau levels and mitigate tau-induced neurodegeneration in cultured cells, Drosophila, and mouse models (Iijima et al., 2010; Ando et al., 2016).

Considering the crucial impact of tau kinases and tau phosphatases on the abnormal hyperphosphorylation of tau in the brains of patients with AD, we further examined the protein levels of tau kinases and tau phosphatases. Microtubule affinity-regulating kinase (MARK) (Drewes et al., 1997; Nishimura et al., 2004; Sun et al., 2016), glycogen synthase kinase (GSK)-3β, Cyclin-dependent Kinase 5(CDK5), and Protein Phosphatase 2A (PP2A) play a significant role in regulating the levels of tau phosphorylation (Baumann et al., 1993; Pei et al., 1997; Gong et al., 2000; Noble et al., 2003; Liu et al., 2016). Our data suggest that CDK5, GSK3β, and PP2A are probably not responsible for the reduction of Tau-Ser262 phosphorylation in dl-NBP-treated P301S mice. Nonetheless, it is noteworthy that we uncovered that the level of MARK4 increased in P301S mice, compared with WT mice. Simultaneously, the protein level of MARK4 significantly reduced in dl-NBP-treated P301S mice, compared with vehicle-treated P301S mice. Since MARK4 is closely related to hyperphosphorylation at Ser262 in AD mice, our results thus suggest that dl-NBP could target the tau kinase MARK4 and, in turn, result in the downregulation of hyperphosphorylation tau at the site of Ser262. Further studies are warranted to investigate the involvement of other kinases, such as PKA, P38, ERK, and JNK, in dl-NBP-mediated neuronal protective role in AD.

Neuroinflammation has also been reported to play a significant role in AD (Cras et al., 1991). In addition, dl-NBP has been shown to play an anti-inflammatory role in ischemic animal models (Mcgeer and Mcgeer, 2010; Meraz-Rios et al., 2013; Ossola et al., 2016). Thus, we tested the levels of inflammatory factors in dl-NBP-administered mice and observed that AD mice treated with dl-NBP exhibited lower protein levels of IL-1β, TNF-α, and GFAP, compared with vehicle-treated AD mice. At the same time, the immunoreactivity of CD11b and GFAP, markers of microglia and astrocytes, respectively, significantly reduced in the brains of dl-NBP-treated P301S mice, compared with control P301S mice. Our results thus suggest that dl-NBP plays an anti-inflammatory role in the P301S mouse model.

## Conclusion

Our study provides experimental support for the protective effect of dl-NBP in P301S mice. The administration of dl-NBP markedly reduced tau phosphorylation levels at Ser262, improved memory and learning, and ameliorated synaptic deficits in AD mice. In addition, treatment with dl-NBP also decreased the inflammatory response in AD mice. Taken together, these new findings suggest the potential of dl-NBP as a treatment option for AD and other tauopathies.

## Data Availability Statement

The original contributions presented in the study are included in the article, further inquiries can be directed to the corresponding author.

## Ethics Statement

The animal study was reviewed and approved by the Institutional Animal Care and Use Committee at Tongji Medical College, Huazhong University of Science and Technology.

## Author Contributions

GL designed the study. YC, YY, and JH carried out the experiments and performed the measurement. YW, XJ, and LL analyzed the data. YC drafted the manuscript. GL, RM, and ZW modified the manuscript. All authors read and approved the final manuscript.

## Conflict of Interest

The authors declare that the research was conducted in the absence of any commercial or financial relationships that could be construed as a potential conflict of interest.
