# Corrigendum: Dl-3-n-Butylphthalide Reduces Cognitive Deficits and Alleviates Neuropathology in P301S Tau Transgenic Mice

**DOI:** 10.3389/fnins.2021.716049

**Published:** 2021-06-25

**Authors:** Yanmin Chang, Yi Yao, Rong Ma, Zemin Wang, Junjie Hu, Yanqing Wu, Xingjun Jiang, Lulu Li, Gang Li

**Affiliations:** ^1^Department of Neurology, Union Hospital, Tongji Medical College, Huazhong University of Science and Technology, Wuhan, China; ^2^Department of Pharmacology, Tongji Medical College, Huazhong University of Science and Technology, Wuhan, China; ^3^Harvard Medical School, Boston, MA, United States

**Keywords:** Dl-3-n-butylphthalide, tau, P301S, cognitive deficits, neuroinflammation

In the published article, there was an error in affiliation 1. Instead of “Tongji Medical College, Wuhan Union Hospital, Huazhong University of Science and Technology, Wuhan, China,” it should be “Department of Neurology, Union Hospital, Tongji Medical College, Huazhong University of Science and Technology, Wuhan, China.”

Additionally, there was an error in affiliation 2. Instead of “Tongji Medical College, Huazhong University of Science and Technology, Wuhan, China,” it should be “Department of Pharmacology, Tongji Medical College, Huazhong University of Science and Technology, Wuhan, China.”

Lastly, the affiliation superscript was missing for author Gang Li. This author should be affiliated to affiliation 1—“Department of Neurology, Union Hospital, Tongji Medical College, Huazhong University of Science and Technology, Wuhan, China.”

The authors apologize for these errors and state that they do not change the scientific conclusions of the article in any way. The original article has been updated.

